# Collaboration leads to cooperation on sparse networks

**DOI:** 10.1371/journal.pcbi.1007557

**Published:** 2020-01-21

**Authors:** Simon D. Angus, Jonathan Newton

**Affiliations:** 1 Department of Economics, Monash University, Melbourne, Australia; 2 SoDa Laboratories, Monash Business School, Monash University, Melbourne, Australia; 3 Institute of Economic Research, Kyoto University, Kyoto, Japan; University of California Irvine, UNITED STATES

## Abstract

For almost four decades, cooperation has been studied through the lens of the prisoner’s dilemma game, with cooperation modelled as the play of a specific strategy. However, an alternative approach to cooperative behavior has recently been proposed. Known as collaboration, the new approach considers mutualistic strategic choice and can be applied to any game. Here, we bring these approaches together and study the effect of collaboration on cooperative dynamics in the standard prisoner’s dilemma setting. It turns out that, from a baseline of zero cooperation in the absence of collaboration, even relatively rare opportunities to collaborate can support material, and robust, levels of cooperation. This effect is mediated by the interaction structure, such that collaboration leads to greater levels of cooperation when each individual strategically interacts with relatively few other individuals, matching well-known characteristics of human interaction networks. Conversely, collaboratively induced cooperation vanishes from dense networks, thus placing environmental limits on collaboration’s successful role in cooperation.

## Author summary

It is traditional in game theory to model cooperation as the play of a given strategy in a social dilemma. This approach is subject to the criticism that cooperation has to be separately defined for each new situation in which it is considered. Recently, collaboration—the ability to participate in collective decision making and optimization, has been proposed as an alternative approach to cooperative behavior. Collaboration has the benefit that it can be defined independently of any game. We bring these two approaches together, showing that even relatively rare opportunities for collaboration can support robust levels of cooperation, especially when interaction networks are sparse. This result is significant as human networks are often sparse and so our results support the wide distribution and persistence of cooperation across human populations.

## Introduction

It is generally accepted that cooperation, understood in a broad sense, is widespread amongst great apes [1] and there is evidence that humans are more cooperative, in the sense of being more likely to undertake jointly intentional behavior, than other great apes [2, 3]. This has led to the conjecture, known as the shared intentionality hypothesis [4] or the Vygotskian intelligence hypothesis [5–7], that the particularly social and cooperative nature of humans provided a niche in which sophisticated cognitive capacities could evolve. A formal model of this conjecture has been given recently by dos Santos and West [8].

Following Axelrod and Hamilton [9], cooperation has usually been modeled as playing *Cooperate* rather than *Defect* in a prisoner’s dilemma (Table 1). As noted in Newton [10], all evolutionary models that work in favor of this type of cooperation rely on inducing positive assortativity in behavior. That is, for playing Cooperate to be profitable, it must be played a disproportionate amount of the time against Cooperate. Mechanisms to induce such assortativity include repeated interaction [11], kin-selection [12, 13], partner choice [14, 15] and group selection [16–18], a particular case of the latter being imitative dynamics in which players who obtain high payoffs from playing cooperate against other cooperators are imitated [19]. For a concise and unified description of such mechanisms, the reader is referred to Nowak [20], for an extensive and detailed discussion of cooperation, to Bowles and Gintis [21], for a specialized review of parochial altruism theory, to Rusch [22]; or to other key studies [23–29].

**Table 1:** Prisoner’s dilemma giving payoffs to interactions, 2*b* > *c* > *b* > 0. Entries are interaction-payoffs of an individual whose strategy is given by the row when interacting with an individual whose strategy is given by the column. Cooperation provides a benefit of *b* to both players, but costs *c* to the cooperating player. This payoff specification corresponds to a public goods setting, in which paying a cost of *c* provides a public good worth *b* to each player. An equivalent interpretation is gift giving, in which a cost c* = *c* − *b* can be paid to provide a gift worth *b* to the opposing player.

	Cooperate	Defect
**Cooperate**	2*b* − *c*	*b* − *c*
**Defect**	*b*	0

This standard approach to cooperation has paid dividends but is limited in its applicability. Each new environment, typically modeled by a game, requires a cooperative strategy to be defined. In general, however, it is unclear what kind of behavior should be considered cooperative. If Alice altruistically helps Bob to supplant Colm as president of their karate club, is that cooperative? What if both Alice and Bob benefit from supplanting Colm? To address a diversity of situations, an alternative game theoretic approach to cooperative activity has recently been proposed. This is based on *mutualism* [30–32] and is referred to as *collaboration* to distinguish it from the existing approach [33]. A set of individuals is said to collaborate if its members adjust their strategies to their mutual benefit. To emphasize, collaboration is defined as a type of decision making, in contrast to cooperation, which is defined as a strategy.

The factors that work for or against the evolution of collaboration have been studied in a variety of environments. Angus and Newton [33] consider collaboration in coordination games and study a group selection model in which the number of collaborators in a population affects the speed of cumulative techno-cultural gains. Newton [34] considers the evolution of collaboration across a broad range of environments and gives conditions under which positive amounts of collaboration can be expected to evolve. Most recently, Rusch [35] gives a comprehensive study of collaboration in two player, two strategy games, showing that amongst such games, the prisoner’s dilemma is the most hostile to the evolution of collaboration, but that collaboration can evolve even in *niches* (mixtures of games) in which the prisoner’s dilemma makes up as much as forty percent of all interactions.

The current study brings together these two literatures and directly considers the impact of collaboration on cooperation. Specifically, we consider the impact of mutualistic decision making by small groups on cooperative outcomes in structured populations when the payoffs from pairwise interactions are given by a prisoner’s dilemma. Strategies are updated according to coalitional better response dynamics [36–38] that in the absence of collaboration reduce to the classic better response dynamics that underpin the concept of (Cournot-)Nash equilibrium [39, 40]. Under these dynamics (and in stark contrast to imitative dynamics), any individual that updates his strategy, either alone or as part of a coalition, will obtain a (weakly) higher payoff after the update than he obtained before.

Under purely individualistic decision making, such dynamics lead to zero cooperation. This is true on any interaction structure and arises simply from the fact that defection is a strictly dominant strategy in the prisoner’s dilemma. From this baseline of zero cooperation in the absence of collaboration, cooperative behavior increases in the frequency of collaborative decision making relative to individualistic decision making. This effect is mediated by the interaction structure, so that collaboration leads to greater levels of cooperation when each individual in the population interacts with relatively few other individuals. As the density of the graph of interactions increases, collaboration is less effective at inducing cooperation. In the limit of uniform interaction across a population, small amounts of collaboration fail to lead to significant amounts of cooperation. Notably, empirical studies have found human social networks to be sparse [41–43].

These findings are consistent with the experimental literature on the effect of communication in social dilemmas played by humans [44, 45], which provides strong evidence that if players are allowed to communicate by message or speech, then they use the opportunity to collaboratively choose their strategies. This leads to much higher rates of cooperation than in the absence of communication. The reader is referred to Balliet [44] for a survey of this literature that goes all the way back to Deutsch [45].

Importantly, the decision making unit in our model is not fixed. Individualistic decision making always coexists with any level of collaboration that we consider. If Alice and Bob, who have been defecting against one another, collaborate to play Cooperate to their mutual benefit, then their decision making is collective. This does not stop them from making individual decisions in future, and when Alice is called upon to make a decision as an individual, she will play Defect, as this is her optimal action from an individual perspective, no matter what Bob is doing. The frequency of individual decision making compared to collective decision making is then an empirical question, the answer to which will determine the frequency of cooperation.

Finally, we emphasize that the purpose of this paper is not just to give another mechanism by which cooperation can evolve. The authors regard the question of whether people may play Cooperate in prisoner’s dilemmas, while interesting, as being of less importance than collaboration, which is a comprehensive and multipurpose faculty. As such, our main goal is to make a bridge between an old literature—cooperation, and a relatively new literature—collaboration, by considering in what circumstances the former can arise as a side effect of the latter.

## Model

We summarize here the model. Details can be found in S1 Text. Following Bowles [18], consider a population of size *n* = 32 individuals. Time is divided into an initialization period *t* = 0 and *t* = 1, …, *T* periods of strategic updating.

### Population structure

The interaction structure within the population is given by an undirected graph on the set of individuals. This is determined at *t* = 0 and does not change thereafter. The graph is parameterized by its edge density *e*, which equals the ratio of the number of edges in the graph to the number of possible edges. Our main treatments consider Erdös-Renyi random graphs and it is shown in the S1 Text that results are similar for Watts and Strogatz [46] small worlds. The graph connects each individual to those with whom he has a high degree of interaction such as relatives, friends and hunting partners [23, 47, 48]. If there is an edge between two individuals, we say that they are *neighbors*.

### Strategies and payoffs

At any one time, any given individual plays one of two strategies, Cooperate or Defect. His payoffs are determined from playing the prisoner’s dilemma in Table 1 against each of his neighbors on the interaction graph. Thus, he obtains an interaction-payoff from his interactions with each of his neighbors. For example, if he plays Cooperate and a given neighbor plays Defect, then he obtains a payoff of *b* − *c* from his interaction with that neighbor. His overall payoff is then the sum of these interaction-payoffs over all of his neighbors.

### Strategy updating

At *t* = 0, each individual in the population is randomly assigned a strategy. In our main treatment, half of the population is assigned Cooperate and the other half is assigned Defect. In the S1 Text we show that results are robust to initial conditions in which every individual plays Cooperate and every individual plays Defect respectively.

**Fig 1:**
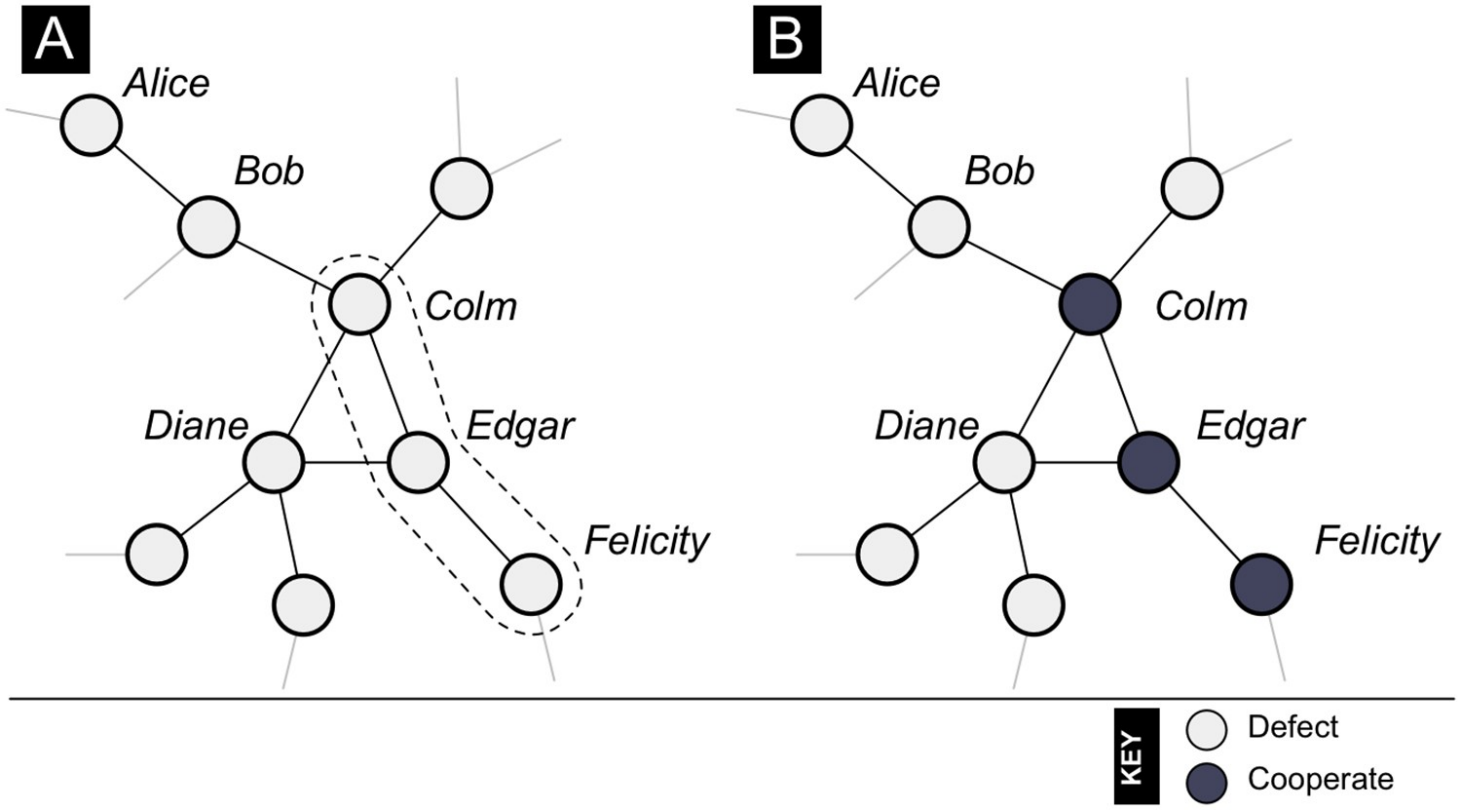
Collaborating to cooperate. Vertices indicate players, edges indicate interactions. **Panel A**: The graph restricted to the set of individuals {*Colm*, *Edgar*, *Felicity*} (dashed line) constitutes a connected sub-graph and so these individuals can occasionally collaborate in updating their strategies when it is profitable to do so. This is not the case for the set of individuals {*Alice*, *Bob*, *Diane*} as the graph restricted to this set is not connected. **Panel B**: Strategies following a collaborative decision by {*Colm*, *Edgar*, *Felicity*} to switch to the strategy Cooperate. Such a switch would be a coalitional better response at *b* = 4, *c* = 5, but not at *b* = 3, *c* = 4, as in the latter case Colm would lose payoff from such a switch.

Strategies are updated by single individuals (*k* = 1) but also by *coalitions* containing $k=2,\ldots ,\bar{k}=5$ collaborating individuals. A set of individuals can only collaborate if the induced subgraph restricted to those individuals is connected (Fig 1A). That is, within a given coalition, players interact with one another either directly or indirectly. In the S1 Text we show that similar results obtain when we restrict coalitions to be *cliques* of individuals in which every individual in the coalition is a neighbor of every other individual in the coalition.

Every period, *t* = 1, …, *T*, a coalition (that may be a single individual) is randomly selected to update strategies. The probability of the selected coalition containing *k* individuals is given by the probability of *k* − 1 successes when drawing from a binomial distribution with success probability *p*. Thus *p* parameterizes the distribution over the size of the updating coalition and, consequently, the frequency of collaboration. If *p* = 0 then there is no collaboration and only individuals update their strategies. If *p* > 0 then there is some level of collaboration.

The updating coalition plays a *coalitional better response*, adjusting the strategies of its members so that by doing so every member of the coalition obtains payoffs at least as high as their current payoffs, holding the strategies of all the other individuals fixed (Fig 1B) [33, 37, 38, 49]. Specifically, consider the possibilities (i) if every member of the coalition simultaneously switches to Cooperate, then every member of the coalition obtains payoffs at least as high as his current payoffs, and (ii) if every member of the coalition simultaneously switches to Defect, then every member of the coalition obtains payoffs at least as high as his current payoffs. If (i) but not (ii) holds, then every member switches to Cooperate. If (ii) but not (i) holds, then every member switches to Defect. If (i) and (ii) hold, then each of these two outcomes occurs with equal probability. If neither (i) nor (ii) hold, then every member of the coalition maintains his current strategy.

## Results

**Fig 2:**
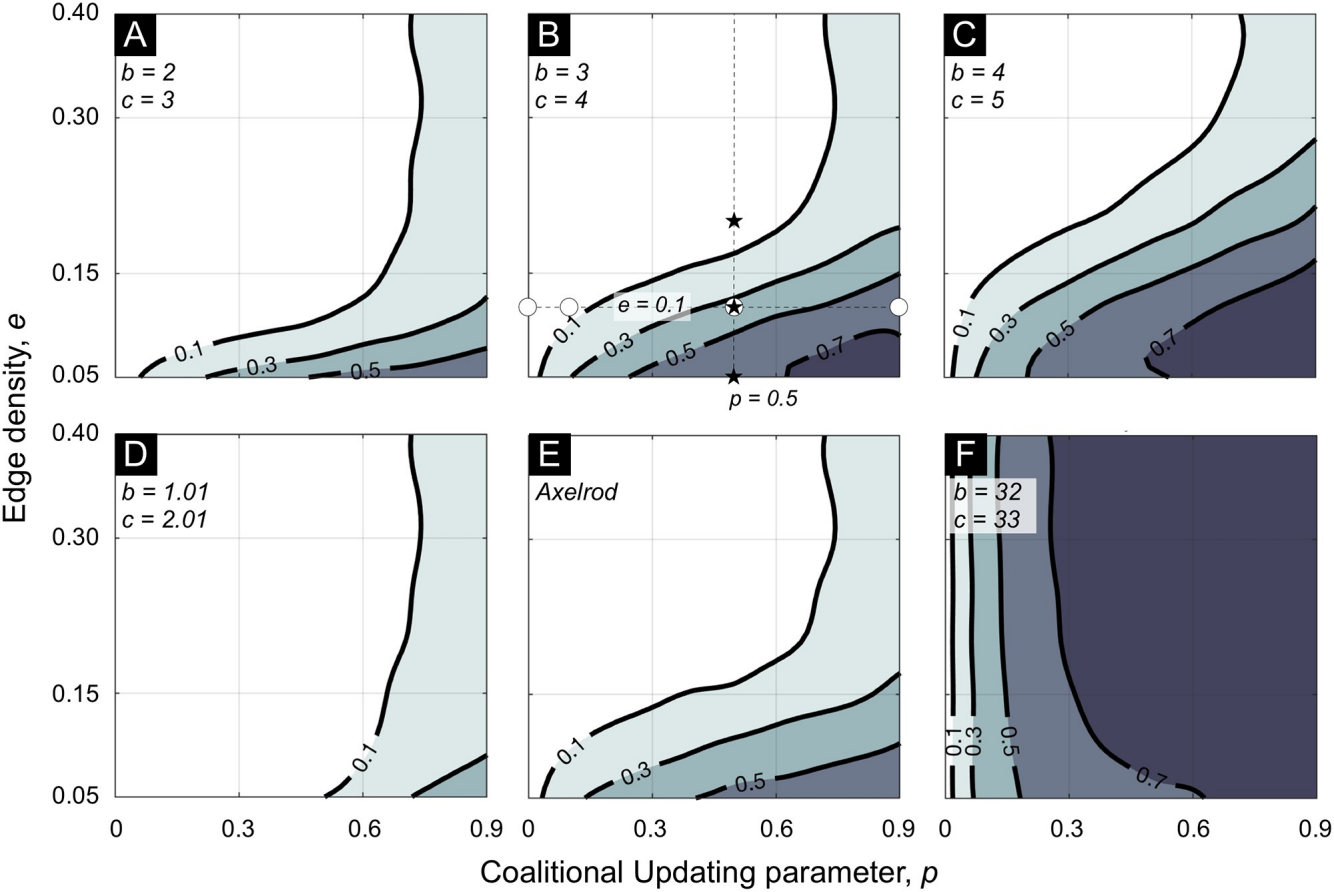
Cooperation by level of collaboration and interaction structure. Contours and shading indicate average share of cooperation in the population over updates 2, 501 to 3, 000 of the model run over each of 50 different random graphs, for given edge density *e* and collaboration parameter *p*. Panels **A**–**D**, and **F** present simulation outcomes at indicated values of *b* and *c* (see Table 1). Benchmark conditions are given in panel B: circle and star markers indicate positions in (*p*, *e*) space at which experiments reported in Fig 3 (circles) and Fig 4 (stars) are run. Simulation results under the payoffs of [9] (i.e. payoffs 3, 0, 5, 1) are presented in panel **E** for comparison. Panel **D** presents the boundary case in which an individual will only collaboratively switch from Defect to Cooperate if all of his neighbors will play Cooperate after the switch. Panel **F** presents the boundary case in which an individual will always collaboratively switch from Defect to Cooperate if at least one of his neighbors switches with him.

Results are summarized in Fig 2. There is no cooperation in the absence of collaboration (*p* = 0) at any edge density (*e*). Cooperation increases in collaboration and does so faster at lower edge densities. For low edge densities, cooperation levels in excess of 70% are observed at high levels of collaboration under some treatments. For a fixed positive level of collaboration (*p* > 0), cooperation decreases in edge density. These results are robust to the variations in network structure (Erdös-Renyi or Watts-Strogatz), population size, initial conditions (zero, half or full cooperation at *t* = 0), and coalition formation (connected subgraphs or cliques) noted in our description of the model.

The relationship between cooperation and *p* is non-linear and varies qualitatively across treatments. In Fig 2F, for example, it can be seen that for low edge densities (e.g. *e* = 0.15), the marginal effect of increased *p* on cooperation is higher at small values of *p*. In Fig 2C, it can be seen that for high edge densities (e.g. *e* = 0.30), the marginal effect of increased *p* on cooperation is higher at large values of *p*.

**Fig 3:**
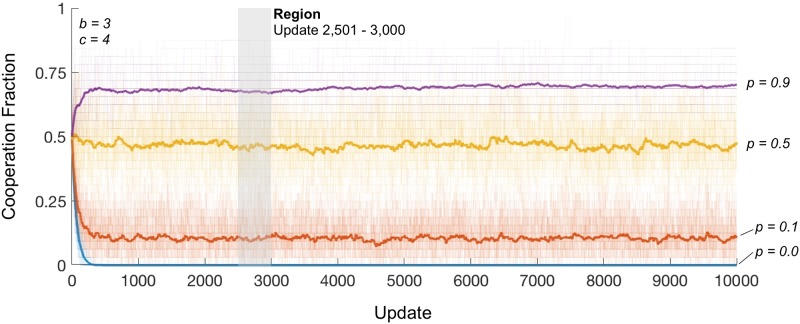
Cooperation under collaboration in the long run. Average share of cooperation in the population (thick lines) under different collaboration parameter values, p, as indicated, over 50 different random-graphs having *e* = 0.10 and benchmark conditions. Coloured, faint lines show share of cooperation in the population from each run. Region indicated (grey transparency) corresponds to updates used to calculate average cooperation fraction for contour plots in Fig 2.

As can be observed in Fig 3 for the parameter values marked by circles in Fig 2B, the amount of cooperation within a population can show substantial volatility over time, especially at intermediate levels of *p*. This is because, at intermediate levels of *p*, there is a reasonably high probability of updating by both (i) large coalitions of players, in which each player has a considerable proportion of his neighbors within the coalition, who can profitably switch from Defect to Cooperate, and (ii) small coalitions (including the case of a single updating individual) that can profitably switch from Cooperate to Defect. The interplay of these two effects leads to rise and fall of the observed proportion of cooperating individuals in the population. An implication of the dynamics described above is that cooperation arises at predictable locations. Specifically, it arises at locations on the graph where highly connected groups of players can form coalitions.

However, despite the intertemporal volatility of cooperation within given simulations, the average amounts of cooperation across simulations (as reported in Fig 2) converge rapidly, the residual variance being immaterial to our results. Furthermore, we see in the S1 Text, that if collaboration is turned off for 1000 periods, perhaps due to some catastrophic trust-reducing event, then cooperation rapidly reduces to zero, but that when collaboration restarts, cooperation rapidly returns to the levels shown in Fig 3.

**Fig 4:**
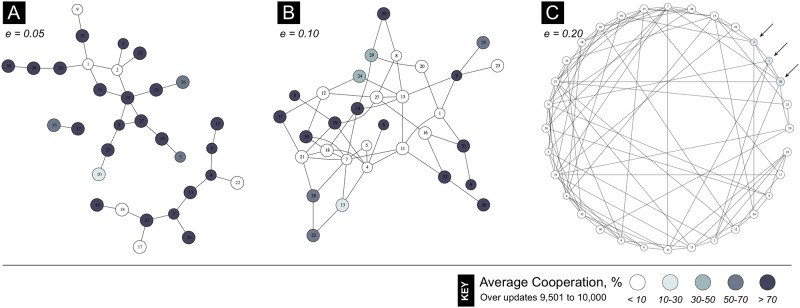
Collaboration fosters cooperation on sparse networks. Percent of time that individuals in the population played Cooperate across updates 9,501 to 10,000, with colouring as per the key at bottom of figure. Presentation is for a single run over each of three example networks with differing edge density *e*, as given in panels **A**–**C**, at benchmark conditions (*b* = 3, *c* = 4) and collaboration parameter *p* = 0.5. Note that in panel **A** (*e* = 0.05) the network is disconnected, being comprised of three components; and in panel **C**, only three agents (indicated by arrows) cooperated significantly (10 − 30%) over the sample updates.

For a fixed level of collaboration (*p* = 0.5), Fig 4 illustrates the effect of increasing edge density with some example graphs from the treatments marked by stars in Fig 2B. For low edge densities, it is more likely that collaborating individuals comprise a high proportion of one another’s neighbors. Consider the clique of three players in Fig 4A. If these individuals are currently playing Defect, then they all gain from collaborating and switching to play Cooperate, regardless of the strategies played by other individuals. In denser graphs, such as in Fig 4C, individuals are likely to have a high proportion of their neighbors outside of any such clique, so collaborative switches to Cooperate are unlikely to be profitable.

Note that there is no conflict between the observations above that (i) connectivity within a coalition encourages switches from defection to cooperation, and (ii) connectivity between individuals within a coalition and individuals outside the coalition discourages switches from defection to cooperation. In fact, the key factor in determining whether such a switch is profitable for any given player within the coalition is the proportion of that player’s neighbors that are also in the coalition. Hence, a high level of connectivity within a coalition is good for cooperation, but a high level of connectivity between a coalition and individuals outside of the coalition is bad for cooperation.

The emergence of cooperation in relatively dense subgraphs that are relatively isolated from the rest of the graph is consistent with results that derive from some individualistic models of cooperation in the literature [50]. However, in a collaborative model there is the added complexity that potential coalitions may be nested within potential coalitions. That is, if a potential coalition *S* has dense internal connections, then this can correspond to dense external connections for some other potential coalition *T* ⊂ *S*. Thus the very factor that makes *S* a good candidate for collaborating to cooperate can make *T* a bad candidate. See [38, 51] for an analysis of such graph theoretical considerations in coordination games.

## Discussion

Collaborative choice in any game is mutualistic: all parties gain from adjusting their strategies together. Cooperation in prisoner’s dilemmas is altruistic. This study has examined circumstances in which collaboration in choice leads to cooperation in behavior. That is to say, in this setting, altruism in behavior emerges as a consequence of mutualism in decision making. This separation of decision making and behavior, while easy to comprehend, is missing from much discussion of cooperation. The authors believe there is much to be gained from paying careful attention to this distinction.

It is instructive to relate the emergence of cooperation under collaboration to the rules for the evolution of cooperation categorized in Nowak [20]. Although collaboration is defined independently of the strategy set, we can consider the particular case in which a set of individuals, all of which currently play Defect, switch to Cooperate. In this case, the simultaneous switch by all of these individuals to Cooperate can be seen as an instantaneous form of *direct reciprocity*, one of the five rules of the cited paper [20]. Specifically, as with direct reciprocity, such a switch is profitable for all coalition members precisely because they all switch. Furthermore, as the collaborating players form a connected subgraph, each individual in the set tends to have a higher than average proportion of their interactions with other individuals in the set. This is *network reciprocity*, another of the five rules of Nowak [20]. For fixed coalition size, this effect decreases in the density of the graph, leading to a decrease in cooperation.

Note that the comparison that we have just made is specifically for the case of a set of individuals that all switch from Defect to Cooperate. This helps to emphasize the mutualism at the core of collaboration as a concept. If we instead consider a coalition comprised of two neighbors, Alice who is currently playing Cooperate and Bob who is currently playing Defect, then that coalition will never adjust its strategies as part of a coalitional better response, as any alternative strategies for the pair would lead to a lower payoff for Bob.

Further note that the opposition of collective incentives and individual incentives in prisoner’s dilemmas is very stark. The two player prisoner’s dilemma is an extreme situation in that the answers to the questions *‘what should we do?’* and *‘what should I do?’* are always diametrically opposed. This makes the prisoner’s dilemma ideal for demonstrating the power and the limits of collaboration. Other games, in which this opposition is not so strong, may be fertile grounds for exploring further implications of collaborative choice.

Finally, the model of the current paper suggests a potential future avenue for the evolution of collaboration literature. Specifically, the parameter *p* could be considered to vary across individuals and be subject to evolutionary pressure. That is, we could have a *p*_*i*_ for each player *i*. Existing studies [33–35] focus on a discrete trait whereby any given player either can (*p*_*i*_ = 1) or cannot (*p*_*i*_ = 0) participate in collaboration.

## Supporting information

S1 Text Supporting information. Supporting model details, figures and robustness results.
(PDF)
