# Supplementary material for: Collaboration leads to cooperation on sparse networks
Source: PLoS Comput Biol. 2020 Jan 21;16(1):e1007557. doi: 10.1371/journal.pcbi.1007557 (PMC6974046; doi:10.1371/journal.pcbi.1007557)
Supplement: S1 Text — Supporting model details, figures and robustness results. (PDF) [file pcbi.1007557.s001.pdf]

Collaboration leads to cooperation on sparse networks

Angus & Newton, 2019

S1 Supporting Information

Contents

1 Model: details **1**

1.1 Building the graph and coalition library, $\mathfrak{G}$ 1

1.2 Choosing a coalition $S \in \mathbf{S}$ 2

1.3 Run-time Coalition Formation (large n only) 3

2 Strategic switching: details **3**

3 Robustness **5**

3.1 Extreme initial conditions 5

3.2 Disruption to the ability to collaborate 5

3.3 Small-world interaction graphs 5

3.4 Coalitions as cliques 5

3.5 Large n 7

3.6 Costly Coalitions 8

1 Model: details

Pseudocode for the running a single replicate of the main numerical model is given in Alg 1.[1] Each replicate is initiated by setting (and recording) the random seed; importing the graph G and coalitional set $\mathbf{S}$ from the graph and coalition library $\mathfrak{G}|_e$ for the given edge density parameter e; generating the initial strategy vector (50% cooperators); and calculating initial payoffs. Subsequently, the main *update* loop is run T times. This loop involves selecting one coalition (or individual) S to update strategies using the coalitional better response behavioral rule, then re-calculating payoffs. Details on the main steps of the algorithm are given below.

1.1 Building the graph and coalition library, $\mathfrak{G}$

Prior to running simulations, a graph and coalitional library, $\mathfrak{G}$ is created which provides for efficient run-time computation.

Denote the set of individuals by N, with $|N| = n$. A random graph, $G(N, E)$, is constructed with N as its vertex set and E, consisting of unordered pairs of elements of N, as its edge set. At each graph density e and for each of 50 random seeds[2] (seed: $1 \ldots 50$), $G(N, E)$ is formed by first computing the desired number of edges, $(e.\frac{n(n-1)}{2})$ rounded to the nearest integer, then uniformly selecting this number of edges to form E. In a second step, if any isolated individuals remain, such an individual is selected and an edge added at random to some other individual. This is repeated until no isolated individuals remain. Due to this second step, at very low levels of e, the graph density is slightly higher than e. Note, however, that this second step does not guarantee that G is connected.

Next, for each G in the library, all possible coalitions for coalitional sizes $k = 1 \ldots \bar{k}$ are enumerated and stored together with G in $\mathfrak{G}$. Enumeration proceeds iteratively. For $k = 1$ (individualistic updating), the coalitional set $\mathbf{S}_{k=1}$ is simply N. For $k = 2$, the set of feasible coalitions is the set of pairs of individuals

[1] Fully documented code, with examples and routines to replicate the main results in the paper is available for download at http://github.com/specialistgeneralist/Collaborate2Cooperate/.

[2] We use the builtin MATLAB® mt19937ar random number stream (Mersenne Twister with Mersenne prime $2^{19937} - 1$).

Algorithm 1 One simulation replicate (rep) for given parameter values

Require: T, the maximum number of iterations in a simulation; Γ the game-table; $\mathfrak{G}$, the pre-formed graph and coalition library; $n = |N|$, the number of agents in the population; k, the maximum size of a coalition; p the coalitional updating parameter; e the edge density

{*initialise:*}
$seed \leftarrow$ Set and record random seed(rep)
$G, \mathbf{S} \leftarrow$ Import graph and coalitions for this graph($\mathfrak{G}|_e, rep$)
$x \leftarrow$ Initialise strategy vector(n)
$\pi \leftarrow$ Update payoffs(Γ, G, x)
$t \leftarrow 1$
{*update loop:*}
while $(t < T)$ **do**
  $S \leftarrow$ Choose a coalition($\mathbf{S}, k, p$)
  $x \leftarrow$ Obtain better response(Γ, G, S, x)
  $\pi \leftarrow$ Update payoffs(Γ, G, x)
  $t \leftarrow t + 1$
end while

connected by an edge in G, $\mathbf{S}_2 \equiv E$. For $k > 2$, the algorithm proceeds by first creating a stub set from $\mathbf{S}_{k-1}$, then, for each stub, generating a new potential coalition by adding a single additional vertex i that is adjacent to any vertex in the stub. The (unique) list of all newly found coalitions is gathered and forms the coalitional set, $\mathbf{S}_k$, which forms the new stub set for the next iteration of the algorithm. The algorithm continues until the maximum size of k is reached.

Table A: **Summary properties of the graph and coalition library, $\mathfrak{G}$.** All measures given as averages over 50 unique graphs per edge density parameter e.

e	Components	Density	Avg. Degree	Number of size k coalitions $k=1$	$k=2$	$k=3$	$k=4$	$k=5$
0.05	3.3	0.062	1.9	32	31	46	83	162
0.10	1.2	0.10	3.2	32	51	144	476	1,636
0.15	1.0	0.15	4.6	32	74	296	1,348	6,181
0.20	1.0	0.20	6.2	32	99	509	2,887	15,855
0.25	1.0	0.25	7.8	32	124	772	5,098	31,313
0.30	1.0	0.30	9.3	32	149	1,071	7,836	51,371
0.35	1.0	0.35	10.9	32	174	1,398	10,992	74,592
0.40	1.0	0.40	12.4	32	198	1,737	14,302	98,063

Table A provides a tabulation of both graph and coalition size measures for the library $\mathfrak{G}$ used in this work. For very low e, the number of components is larger than 1. Furthermore, the measured average edge density is slightly larger than e due to the process for avoiding isolated vertices.

1.2 Choosing a coalition $S \in \mathbf{S}$

At run-time, each update of the model simulation begins by choosing a coalition S from the coalitional library $\mathbf{S}$ associated with the given graph G. The size of the coalition chosen is parameterized by the coalitional updating parameter p (see Alg. 1). One could apply a uniform selection over all possible coalitions in $\mathbf{S}$, but as shown in Table A, combinatorics dictates that the number of large (e.g. $k = 5$) coalitions greatly exceeds the number of small coalitions, rendering a uniform choice over members of $\mathbf{S}$ skewed strongly in favour larger coalitions. To avoid this, we first determine a coalition size k, $1 \leq k \leq \bar{k}$, according to a discrete probability density function over coalition sizes. Specifically, we use the binomial distribution,

$$P(k|\bar{k}, p) = \binom{\bar{k}-1}{k-1} \cdot p^{k-1}(1-p)^{\bar{k}-k} \, .$$

Table B: Probability of a coalition of size k being selected given coalitional parameter p, given $\bar{k} = 5$.

p	Coalition size				
	$k = 1$	$k = 2$	$k = 3$	$k = 4$	$k = 5$
0.4	0.1296	0.3456	0.3456	0.1536	0.0256
0.5	0.0625	0.2500	0.3750	0.2500	0.0625
0.6	0.0256	0.1536	0.3456	0.3456	0.1296
0.7	0.0081	0.0756	0.2646	0.4116	0.2401
0.8	0.0016	0.0256	0.1536	0.4096	0.4096
0.9	0.0001	0.0036	0.0486	0.2916	0.6561

1.3 Run-time Coalition Formation (large n only)

For combinatorial reasons, when n is large, elucidation of the universe of potential coalitions up to size $\bar{k}$ is computationaly expensive. For large n, we implement a run-time coalition algorithm, replicating the approach to coalition library formation, as follows:

Algorithm 2 Run-time coalition formation algorithm

Require: $\bar{k}$, the maximum size of a coalition; $P(\bar{k})$, the probability distribution over discrete coalition sizes $1 \ldots \bar{k}$; N, the set of agents in the population; and G, the pre-formed $Graph(N, E)$ for this replicate
  {*initialise:*}
  $k \leftarrow$ Choose at random a coalition size($P(\bar{k})$)
  $i \leftarrow$ Choose at random an agent, $i \in N$
  $S \leftarrow i$
  $s \leftarrow |S|$
  $T \leftarrow$ Given G, get adjacent vertices to agents in S, not already in S
  {*main loop (add one other agent at a time):*}
  while $(s < k)$ & $(|T| > 0)$ **do**
    $j \leftarrow$ Choose at random one agent in T
    $S \leftarrow S \cup j$
    $s \leftarrow |S|$
    $T \leftarrow$ Given G, get adjacent vertices to agents in S, not already in S
  end while
  return S

2 Strategic switching: details

In the main paper, the fraction of time that individuals played cooperate over the final 500 updates of a 10,000 update run at benchmark conditions, and $p = 0.5$ is overlaid on the graph structure for differing values of graph density parameter e. We provide here the extant strategic updating dynamics that stands behind these visualisations.

In Figure A we visualise the evolving strategy profile across all 32 individuals and updates 9,501 to 10,000 for each of the three edge density replicates. By inspection, it can be seen that the strategic dynamics comprises common features across each panel: periods of cooperation for two or more individuals are typically created in a coordinated fashion by coalitions $(k > 1)$, whilst defection is either individualistic or enacted by coalitions which comprise a smaller intersection set with a former cooperative coalition. An example of these dynamics is clearly seen playing out for the higher edge density case, $e = 0.20$ (Fig. A, Panel C) as indicated in the figure.

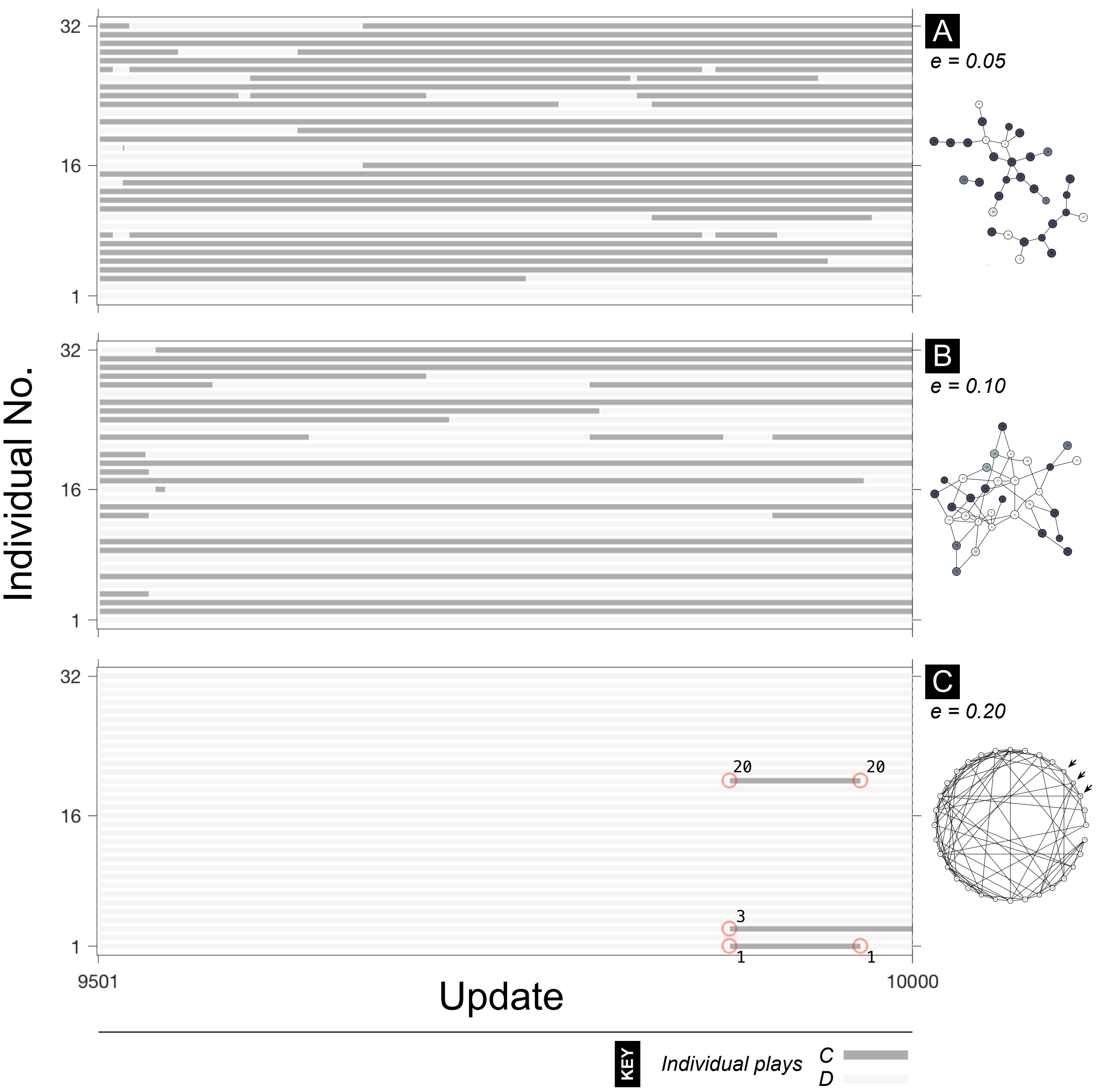

Figure A: **Individual strategy trajectories from the replicates depicted in the network visualisations in Figure 4 of the main paper.** In each panel, the changing strategies of each of the 32 individuals are visualised from left to right over updates 9,501 to 10,000. Darker bands indicate periods of contiguous play of Cooperate, whilst lighter bands indicate periods of contiguous play of Defect. To the right of each of the panels **A**-**C** the associated interaction graph from Figure 4 of the main paper is reproduced. In Panel **C** a coalition of three players $S = \{1, 3, 20\}$ who switch from Defect to Cooperate at update 9,887 is highlighted, together with a subsequent coalition comprising only $S = \{1, 20\}$ who, at update 9,968 switch back to play Defect, leaving individual 3 playing Cooperate on his own.

3 Robustness

To establish the robustness of our main results, we run four robustness exercises as follows.

3.1 Extreme initial conditions

In our benchmark specification, the population is initialized with 50% of the population playing Cooperate and 50% of the population playing Defect. Here we show that this choice of initial population fraction is not material to our results (fig **B**).

3.2 Disruption to the ability to collaborate

In this experiment, we demonstrate that our main results do not exhibit strong dependence on the history of collaborative events. Running the benchmark specification, as in the main paper, after 3000 periods, we effectively 'turn off' the ability to collaborate in the population, by setting the collaboration parameter, p to 0 from period 3001 until period 4000, following which the parameter is returned to its previous level (i.e. 0, 0.1, 0.5, or 0.9). Results of this experiment are given in Figure **C** and demonstrate the lack of sensitivity to such an event.

3.3 Small-world interaction graphs

It has been argued that typical human interaction graphs of given vertex count n and edge density e are characterised by high local clustering and short average path lengths, placing them between the characteristic features of regular lattices on the one hand, and random graphs on the other (Watts, 1999). Consequently, as a robustness check, we test our main results under benchmark conditions, but with Watts-Strogatz 'small-world' graphs (Watts and Strogatz, 1998) instead of the Erdös-Renyi random graphs considered in the main paper.

Following the same methodology as the main paper, we first create a graph-library and pre-enumerate all possible coalitions up to size $k = \bar{k}$. Summary statistics for this library are given in Table **C**.

Table C: **Summary properties of the 'small-world' graph and coalition library, $\mathfrak{G}_{SW}$.** All measures given as averages over 50 unique graphs per edge density parameter e.

e	Components	Density	Avg. Degree	Number of size k coalitions $k=1$	$k=2$	$k=3$	$k=4$	$k=5$
0.05	1.1	0.065	2	32	32	35	39	46
0.15	1.0	0.13	4	32	64	147	371	999
0.20	1.0	0.19	6	32	96	337	1,286	5,169
0.25	1.0	0.26	8	32	128	600	3,002	15,403
0.30	1.0	0.32	10	32	160	935	5,695	34,324
0.40	1.0	0.39	12	32	192	1,331	9,342	62,314

Next, we repeat the benchmark experiments with the 'small-worlds' graph library and present comparison results in Figure **D** below (compare to Figure 2 in the main paper). We conclude that cooperation is slightly more prevalent under small-world interaction structures than it is under Erdös-Renyi random graphs over all parameterizations of the model.

3.4 Coalitions as cliques

We consider an alternative coalition formation process. Specifically, we reduce the benchmark coalitional library to one in which only coalitions which are cliques (complete subgraphs) are included. The aim is to examine an extreme coalition formation framework where every member of a coalition must have a direct

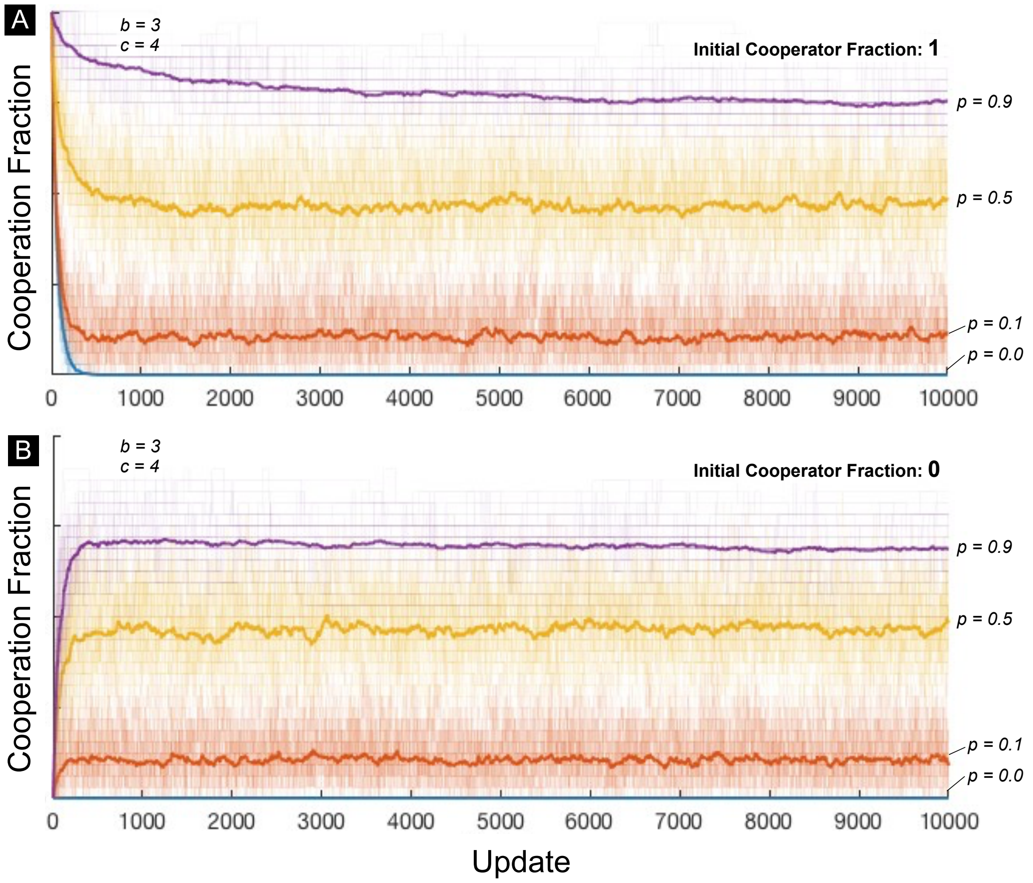

Figure B: **Robustness of long run outcomes to the initial share of cooperation in the population.** Each panel shows the average share of cooperation in the population under benchmark conditions (per the main study), with the initial share of individuals in the population playing Cooperate set to 1 (**A**), or 0 (**B**). (Compare Figure 3 in the main paper.)

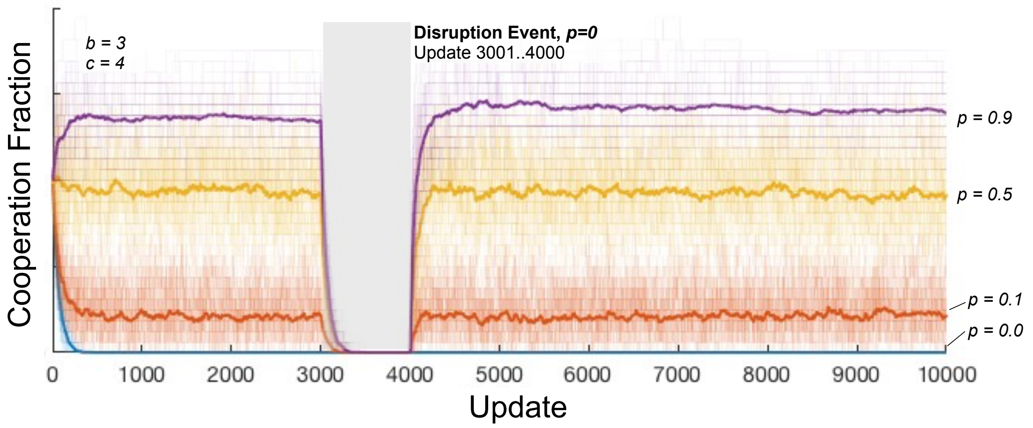

Figure C: **Robustness to collaborative disruption.** Benchmark conditions are used, excepting that, at update 3001, the collaborative probability is suddenly set to $p = 0$, extinguishing all coalitional updating in the model. After 1000 updates, the parameter is set back to its original value for the given experiment. (Compare Figure 3 in the main paper)

payoff-relevant interaction with every other member of the coalition. As such, we consider this setting a more harsh environment for the development of cooperation as large cliques are more common in dense graphs, which our results have shown are not conducive to the collaborative emergence of cooperation.

We present in Table D below the characteristics of the reduced graph library due to the clique restriction.

Table D: **Summary properties of the coalition library when reduced to clique-only (complete sub-graph) coalitions.** All measures given as averages over 50 unique graphs per edge density parameter e.

e	Components	Density	Avg.	Number of size k coalitions				
			Degree	$k = 1$	$k = 2$	$k = 3$	$k = 4$	$k = 5$
0.05	3.3	0.062	1.9	32	31	1	0	0
0.10	1.2	0.1	3.2	32	51	4	0	0
0.15	1	0.15	4.6	32	74	16	0	0
0.20	1	0.2	6.2	32	99	40	2	0
0.25	1	0.25	7.8	32	124	77	8	0
0.30	1	0.3	9.3	32	149	133	25	1
0.35	1	0.35	10.9	32	174	213	66	5
0.40	1	0.4	12.4	32	198	314	141	19

In Figure E we see that, as expected, cooperation is depressed, relative to the benchmark, under the restriction that coalitions be cliques. However, even in this extreme coalitional setting, significant cooperation arises under non-individualistic strategic updating at low to moderate graph densities.

3.5 Large n

All results in the main paper are obtained for $n = 32$ (following Angus and Newton (2015); Choi and Bowles (2007)), here, we consider an enlarged population, $n = 320$.

Given the much larger combinatorial space available at this scale, it is not computational practical to elucidate every possible coalition prior to run-time, and instead, run-time coalition formation is implemented as described in detail above (see Section 1.3).

For comparability, two features of the setup need to be adjusted. First, to ensure that agent-wise update frequency is maintained between the small and large n setting, we run the model for $30,000$ updates, 10 times the benchmark used for $n = 32$. Second, to ensure that payoff trade-offs are isomorphic to the small n case, we re-compute the density parameter, e required to re-produce the equivalent average degree environment produced at the benchmark, $n = 32$ settings[3]. In Table E the large n graph-library properties are given (compare benchmark case, $n = 32$, Table A above).

Table E: **Summary properties of the graph library, $\mathfrak{G}$, with $n = 320$.** All measures given as averages over 10 unique graphs per edge density parameter e.

e	Components	Density	Avg. Degree
0.0059375	14.7	0.0068	2.2
0.01	2.2	0.01	3.3
0.014375	1.1	0.014	4.6
0.019375	1	0.019	6.2

continued over page ...

[3]Note: edge densities of the benchmark conditions would demand a 10x elargement of potential coalition sizes.

Table E: *Summary properties of the graph library, $\mathfrak{G}$, with $n = 320$. (continued)*

e	Components	Density	Avg. Degree
0.024375	1	0.024	7.8
0.029063	1	0.029	9.3
0.034063	1	0.034	10.9
0.03875	1	0.039	12.4

Results of the $n = 320$ case are given below in Figure **F** and reflect the benchmark case reported in the main paper.

3.6 Costly Coalitions

Finally, one can think coalitional updating as introducing a risk that other agents will not follow through with their commitment to update their strategy in a coordinated manner. Here, one may wish to explore a setting in which agents taking part in coalitional switching must pay a switching, or enforcement fee on doing so. Intuition suggests that such a fee would reduce the likelihood of coalitional switching, at the margin, and may depress coalitional play altogether for a sufficiently high coalitional switching cost.

We explore this setting by appyling a one-time switching cost to the payoff calculation for any agent in a coalition (size $k > 1$) selected to update. That is, relative to Alg. 1 we adapt the 'Obtain better response' method, such that an additional argument, switching fee, f is added to the calculation of payoffs for each member of the coalition. Each member pays f once, offsetting the expected outcome of a coalitional strategic update to either C or D.

For example, suppose that $G' = (\{1, 2, 3\}, \{(1, 2), (2, 3)\}$ and all three agents play D. If the two player coalition $S' = \{1, 2\}$ is selected for update and faces coalitional updating cost f, then if $2b - c - f \geq 0$ and $3b - 2c - f \geq 0$ are true respectively for player 1 and 2, a coalitional switch to C would go ahead. Under the benchmark condition, $f = 0$.

Figure **G**(Panels A-E) gives results from 10 random seeds at benchmark conditions of adding $f \in \{1, 2, 4, 8, 16\}$ to the better response decision rule. For low levels of f, results are similar to the benchmark outcome reported in the main paper, however, as expected, as f grows, the region of long-run cooperation diminishes. For prohibitive levels of f, the apparent regions of cooperation are transient in nature only, since the dynamics of coalitional updating has been slowed dramatically by the high cost of coalitional switching. Fig. **G** Panel F gives the average cooperation fraction time-seris confirming the slowed dynamics in this setting.

References

Angus, S.D., Newton, J., 2015. Emergence of shared intentionality is coupled to the advance of cumulative culture. PLOS Computational Biology 11, e1004587.

Choi, J.K., Bowles, S., 2007. The coevolution of parochial altruism and war. Science 318, 636–640.

Watts, D.J., 1999. Networks, Dynamics, and the Small-World Phenomenon. The American Journal of Sociology 105, 493–527.

Watts, D.J., Strogatz, S.H., 1998. Collective dynamics of 'small-world' networks. Nature 393, 440–442.

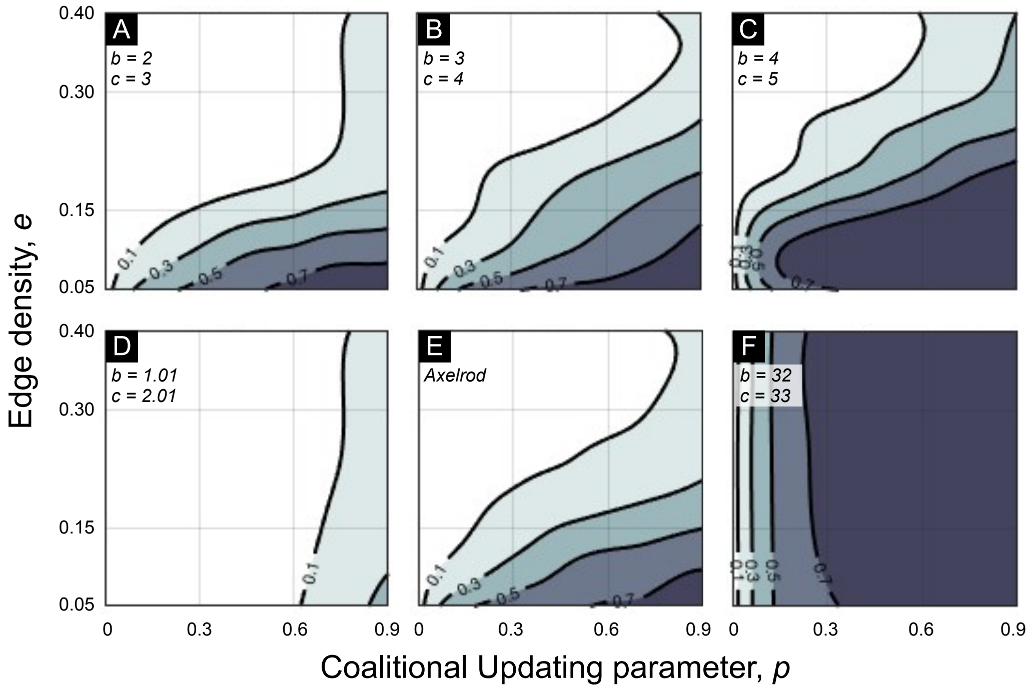

Figure D: **Cooperation by level of collaboration and graph density, 'small-world' graphs**. Experimental results under benchmark conditions as per Figure 2 in the main paper, but with 'small-world' interaction graphs instead of Erdös-Renyi random graphs. (Compare Figure 2 in the main paper.)

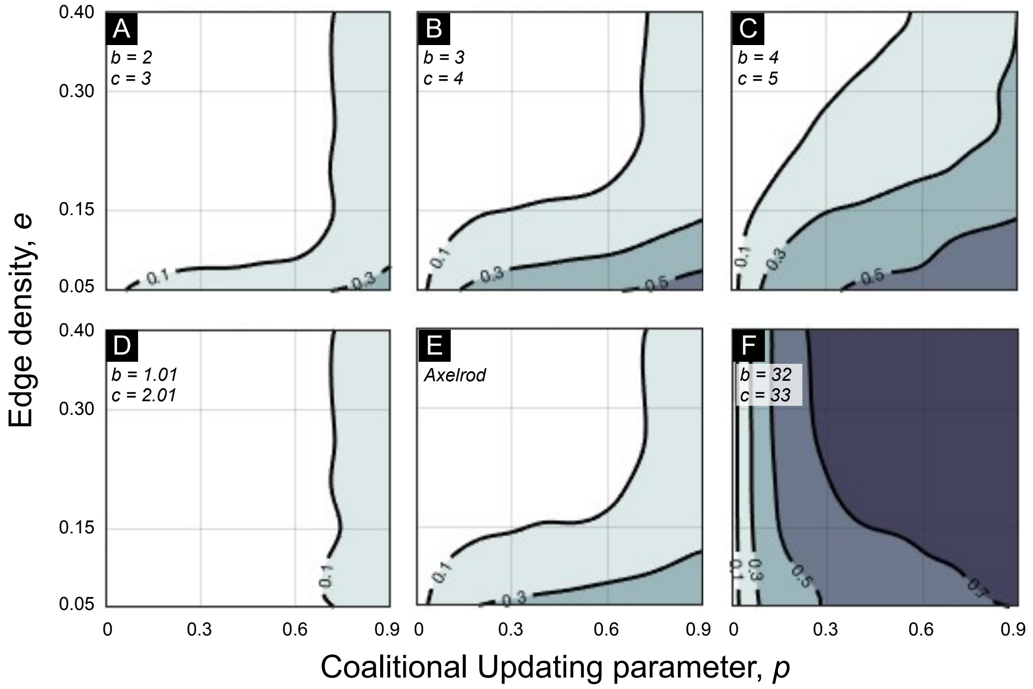

Figure E: **Cooperation by level of collaboration and graph density, clique-only coalition treatment**. Experimental results under benchmark conditions as per Figure 2 in the main paper, but with the coalitional library reduced to cliques (i.e. complete subgraphs). (Compare Figure 2 in the main paper.)

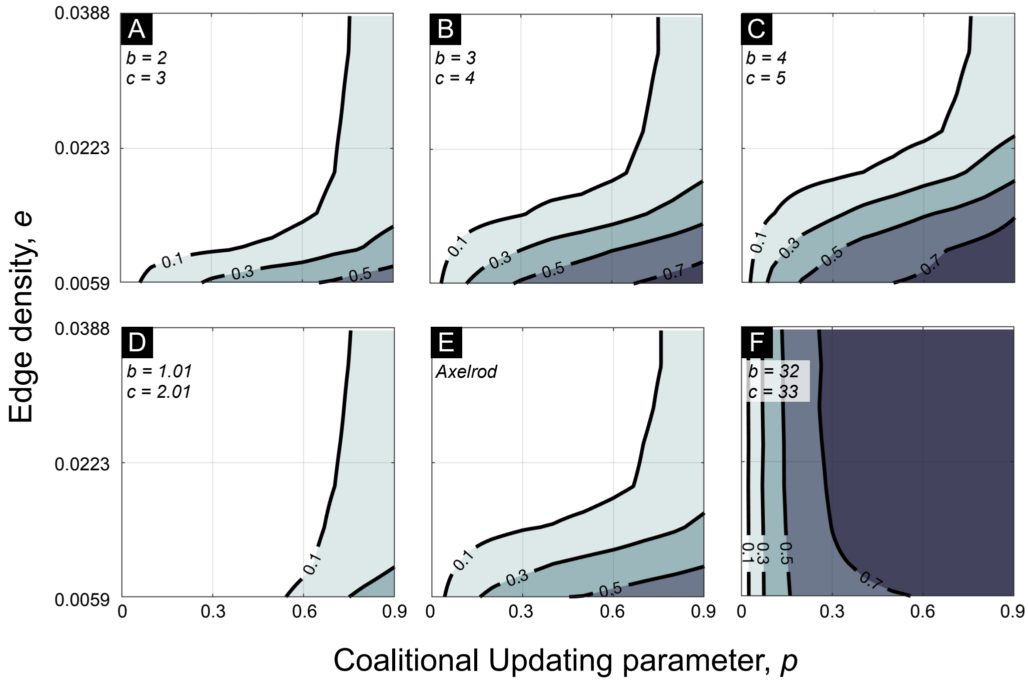

Figure F: **Cooperation by level of collaboration and graph density,** $n = 320$ **study**. Experimental results under benchmark conditions as per Figure 2 in the main paper, but with $n = 320$. (Compare Figure 2 in the main paper.)

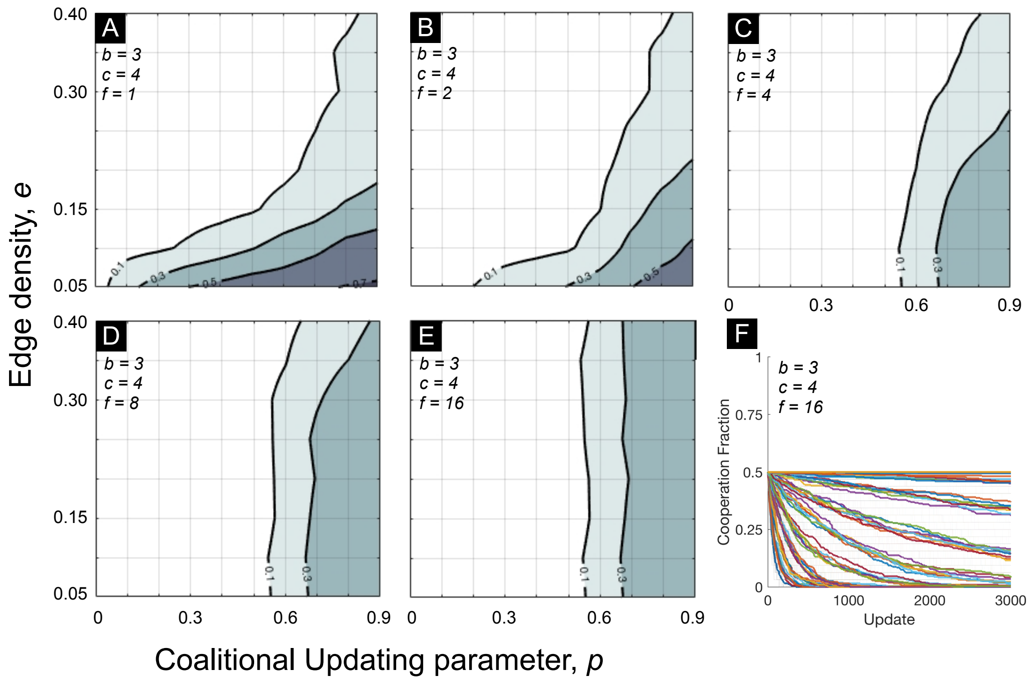

Figure G: **Cooperation by level of collaboration and graph density, costly coalitions study**. Experimental results under benchmark conditions as per A-E: Figure 2 in the main paper, but with coalitional strategic switching fee $f > 0$. (Compare Figure 2 in the main paper.) F: Timeseries of cooperation probablity across 80 different (p, e) experiments with $f = 16$, showing slow convergence dynamics.)
